# SMURF1: a promising target for colon cancer therapy

**DOI:** 10.1093/procel/pwae053

**Published:** 2024-09-27

**Authors:** Xiufang Xiong, Yongchao Zhao, Yi Sun

**Affiliations:** Cancer Institute of the Second Affiliated Hospital, Zhejiang University School of Medicine, Hangzhou 310029, China; Institute of Translational Medicine, Zhejiang University School of Medicine, Hangzhou 310029, China; Department of Hepatobiliary and Pancreatic Surgery, The First Affiliated Hospital, Zhejiang University School of Medicine, Hangzhou 310003, China; Institute of Translational Medicine, Zhejiang University School of Medicine, Hangzhou 310029, China; Cancer Institute of the Second Affiliated Hospital, Zhejiang University School of Medicine, Hangzhou 310029, China; Institute of Translational Medicine, Zhejiang University School of Medicine, Hangzhou 310029, China

## Abstract

**Graphical Abstract:**
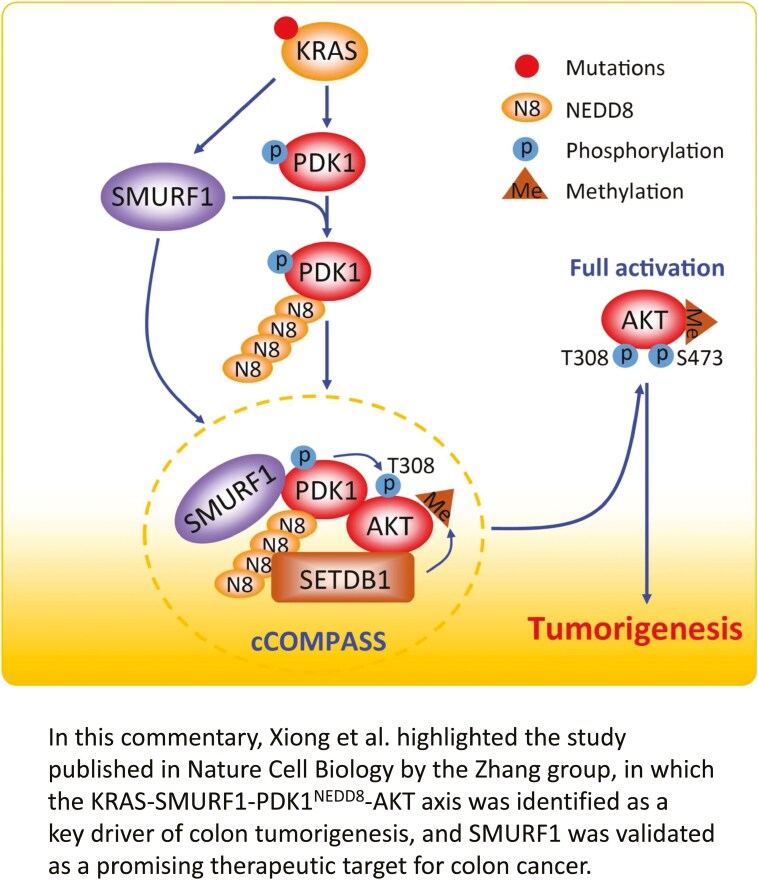

SMURF1 (Smad ubiquitination regulatory factor 1), a HECT-type E3 ubiquitin ligase, plays pivotal regulatory roles in numerous biological processes, encompassing apoptosis, senescence, cell cycle progression, metastasis, and genomic stability, by targeting a variety of key signaling and regulatory proteins for ubiquitylation and proteasome degradation (Fu et al., 2020). Although SMURF1 displays either oncogenic or tumor-suppressing characteristics depending on the cellular context, the majority of studies showed that SMURF1 plays an oncogenic role (Xia et al., 2021). The supporting pieces of evidence include (i) SMURF1 expression is increased in various types of human tissues derived from carcinomas of colon, gastric, and renal clear cells with a positive correlation of poorer patient survival; (ii) SMURF1 knockdown effectively suppresses the growth of colon and gastric cancer cells, and glioblastoma cells in both *in vitro* cell cultures and *in vivo* xenograft models; (iii) SMURF1 inactivation suppresses the migration, invasion, and metastasis of pancreatic and gastric cancer cells, further highlighting its oncogenic role in these contexts (Fu et al., 2020; Xia et al., 2021).

The phosphatidylinositol 3-kinase (PI3K)/AKT signaling pathway serves as a major regulator of cell survival, cell cycle progression, and cell growth. This pathway is often aberrantly activated in various types of human cancers, contributing to the development and maintenance of cancer hallmarks, such as enhanced survival, metastasis, and altered metabolism. Thus, the PI3K/AKT pathway has emerged as a highly promising target for anticancer therapies (He et al., 2021). Fully activated AKT, characterized by phosphorylation at both T308 and S473 sites, serves as a central component of active PI3K/AKT signaling. PDK1 is the only kinase that catalyzes AKT phosphorylation at T308 and is generally acknowledged to be constitutively active due to *in trans* autophosphorylation of its T-loop residue (Casamayor et al., 1999), with substrate accessibility regulation being a key mechanism for controlling PDK1 activity. Given the oncogenic function of AKT, the inhibition of PDK1 would in theory result in the inactivation of AKT, thereby suppressing tumor progression. However, due to its essential physiological functions, the direct inhibition of PDK1 might elicit undesirable side effects (Gagliardi et al., 2018). Therefore, elucidation of the intricate regulatory mechanisms by which PDK1 activates AKT would provide new insights for the development of better therapeutic strategies.

In a most recent study published in *Nature Chemical Biology* (Peng et al., 2024), Peng and his colleagues elucidated a novel mechanism of AKT activation by PDK1 via SMURF1-mediated PDK1 poly-neddylation. Through a systematic mass spectrometry analysis, the authors identified SMURF1 as a novel interacting protein of PDK1. The follow-up study, using paired wild-type and *Smurf1* knockout (KO) MEFs, revealed that Smurf1 deficiency abolished the phosphorylation of Akt at T308 following insulin stimulation. Furthermore, the Smurf1^C426A^ mutant with deficient Nedd8 ligase activity significantly reduced Akt^T308^ phosphorylation, whereas the Smurf1^C699A^ mutant with deficient in ubiquitin ligase activity had no such effect. Furthermore, MEFs derived from Smurf1^C426A^ knock-in mice exhibited a marked impairment in both Pdk1 neddylation and Akt^T308^ phosphorylation upon insulin stimulation. Collectively, these results demonstrated that SMURF1-mediated PDK1 neddylation is crucial for activation of the PDK1-AKT signaling.

Neddylation is a posttranslational modification similar to ubiquitylation, catalyzed by neddylation activating enzyme (E1), neddylation conjugating enzyme (E2) and neddylation ligase (E3) (Zhang et al., 2024). Accumulated data have clearly shown that neddylation pathway is frequently activated with overexpression of all three neddylation enzymes in various types of human cancers, and the inactivation of neddylation pathway suppresses the growth and survival of cancer cells, making it a promising target for anticancer therapies (Zhang et al., 2024). The same Zhang group previously showed that SMURF1 is a neddylation E3 ligase, which promotes SMURF1 self-neddylation to enhance its ubiquitin ligase activity by increased recruitment of ubiquitin E2. Biologically, SMURF1 neddylation promotes tumor growth (Xie et al., 2014).

In this study by the Zhang group, they found that PDK1 is a novel substrate of SMURF1, and the resultant poly-neddylated chain of PDK1 at K163 residue serves as a recognition signal for binding to SETDB1 (SET domain bifurcated histone lysine methyltransferase 1), an oncoprotein overexpressed in many human cancers (Lazaro-Camp et al., 2021), via the N-terminal NEDD8-binding domain of SETDB1. The authors subsequently found that the tri-complex of SMURF1, PDK1, and SETDB1, designated as cCOMPASS (cytoplasmic complex of PDK1 assembled with SMURF1 and SETDB1), facilitates the membrane attachment and T308 phosphorylation of AKT to promote AKT activation (Fig. 1).

**Figure 1.**
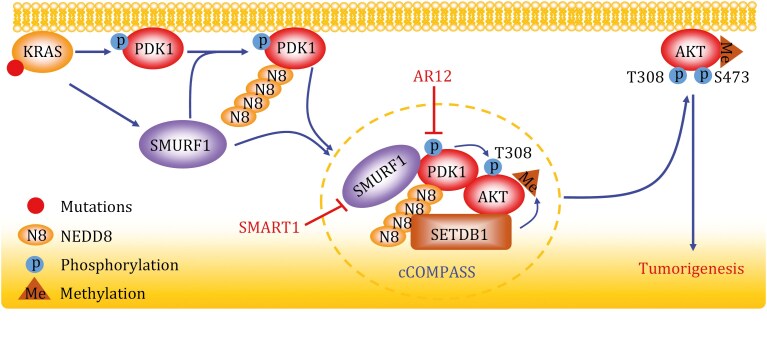
A model for SMURF1 in promoting colon tumorigenesis via the NEDD8-PDK1-SETDB1-AKT signaling pathway. KRAS mutants upregulate SMURF1 transcription, leading to increased SMURF1 levels. Elevated SMURF1 facilitates the poly-neddylation of PDK1, which in turn recruits SETDB1 via the poly-neddylation chains. This recruitment leads to the formation of a cCOMPASS complex comprising SMURF1, PDK1, and SETDB1, which facilitates AKT membrane attachment and T308 phosphorylation for AKT activation. Thus, the KRAS-SMURF1-PDK1-AKT axis acts as a driving force for colon tumorigenesis. Targeting SMURF1 via SMART1 degrader would block this oncogenic axis to suppress tumor growth, and this effect is further enhanced by a PDK1 inhibitor AR12.

Although a previous study has shown that poly-neddylation could act as a posttranslational modification for autophagic degradation of proteotoxic-stress-induced protein aggregates (Ghosh and Ranjan, 2022), the study reported by the Zhang group is the first to show that (i) a poly-neddylation chain could serve as a recognition signal for the recruitment of a specific protein, and (ii) SETDB1 is the first reported “reader” of poly-neddylation chain. Given the fact that many noncullin proteins were poly-neddylated and the neddylation pathway is frequently activated in various types of human cancers (Zhang et al., 2024), it is very likely that SETDB1 might “read” poly-neddylation chain on other cellular proteins. On the other hand, yet-to-be-defined proteins may also act, like SETDB1, as the “reader” of poly-neddylated chains, leading to the assembly of many cCOMPASS-like complexes, as described here, to modulate a variety of biological functions. Future studies in that direction would significantly expand the fields of both neddylation and signal transduction to deepen our current understanding of various biological processes, such as tumorigenesis.

To define the role of the SMURF1-PDK1-AKT axis in the regulation of growth and survival of colon cancer cells and colon tumorigenesis, the authors used *in vitro* cell culture, *in vivo* xenograft tumors and AOM-DSS (azoxymethane-dextran sodium sulfate) chemical carcinogenesis models with approaches including shRNA-based knockdown, small molecular inhibitors, and *Smurf1* KO mice. The results clearly demonstrated that inactivation of this axis has a profound anticancer effect in a manner dependent of SMURF1-induced PDK1 neddylation and subsequent AKT activation. Consistently, the analysis of the TCGA database revealed that SMURF1 is significantly upregulated in colon cancer tissues harboring KRAS mutations, and the high SMURF1 expression is positively associated with poor survival of colon cancer patients. The immunohistochemistry-based analysis of colon cancer tissues showed that elevated SMURF1 levels correlated with poor survival of patients with KRAS mutation. Furthermore, the mutant KRAS transactivates SMURF1 expression and promotes SMURF1-mediated PDK1 neddylation. Finally, a positive correlation among the SMURF1 level, PDK1 neddylation, and AKT phosphorylation in colon cancer tissues was found. Collectively, these results clearly demonstrate that the KRAS-SMURF1-PDK1-AKT axis is a driving force for colon tumorigenesis.

SMURF1 is an enzyme acting as a dual E3 ligase for both ubiquitylation and neddylation, and appears to be an attractive anticancer target. Although the same Zhang group reported previously small molecular inhibitors of SMURF1, such as A01 and A17, with inhibitory activity against SMURF1-mediated SMAD1/5 polyubiquitylation, these compounds had limited effects on tumor cell growth (Cao et al., 2014; Zhang et al., 2017). In this study, the Zhang group, in collaboration with the Rao group, employed a technology known as proteolysis-targeting chimera (PROTAC) (Bekes et al., 2022; Han and Sun, 2023) in an effort to discover a SMURF1 degrader. To this end, the team constructed and screened a structurally diverse PROTAC library through different types of linkers and ligands of E3 ubiquitin ligase and then used the HiBiT technology platform to quickly and quantitatively analyze protein degradation. Through multiple rounds of screening, a CRL4^CRBN^-based, rather-specific small molecule SMURF1 degrader, designated as SMART1 (SMUFR1-antagonizing repressor of tumor 1) was discovered. Given that *Smurf1* total KO mice are vial with a normal lifespan and fertility (Yamashita et al., 2005), targeting SMURF1 for proteasomal degradation by SMART1 might have limited cytotoxicity to normal tissues, which was confirmed to be the case in immune-competent mice.

The effects of SMART1 were carefully evaluated and characterized at multiple levels. Biochemically, SMART1 effectively and selectively degraded SMURF1 protein, but not other family members, including SMURF2 with high homology. Moreover, SMART1 robustly inhibited insulin-mediated PDK1 neddylation, AKT^T308^ phosphorylation, and AKT membrane translocation and activation. Biologically, SMART1 effectively inhibited the growth of colorectal and pancreatic ductal cancer cells harboring KRAS mutations with IC_50_ values ranging from 10 to 50 nmol/L in *in vitro* cell culture settings and remarkably suppressed *in vivo* growth of xenograft tumors derived from mutant RAS-bearing HCT116 cells in a daily dosage of 50 mg/kg administered over a two-week period. Collectively, SMART1 appears to be the first SMURF1 degrader with an effective antitumor activity and low cytotoxicity.

The Zhang group further investigated the combinational effect of SMART1 with AR12, a selective and only ATP-competitive PDK1 inhibitor currently in clinical trials, based upon the observation that NEDD8 conjugation potentially blocked the interaction between AR12 and PDK1. They proposed a “PTM (posttranslational modification) two-hit” therapeutic strategy to concurrently disrupt PDK1 neddylation and phosphorylation. Indeed, such combination showed a significant synergistic effect in both colorectal cancer cell line-derived xenograft (CDX) and patient-derived xenograft (PDX) models. Given the common occurrence of multiple PTMs in most proteins and the potential crosstalk between distinct PTMs, this proposed “PTM two-hit” strategy may offer a promising approach to achieve a more effective target inhibition, thus enhancing therapeutic efficacy.

In summary, this comprehensive study reported by the Zhang group has several novel aspects: (i) SMURF1 is subjected to KRAS upregulation and is a neddylation E3 for PDK1 poly-neddylation; (ii) the poly-neddylated chain on PDK1 can serve as a signal for recruitment of other protein, here SETDB1, which behaves as the first “reader” of the chain. Given that SMURF1 can be self-neddylated (Xie et al., 2014), it is unclear whether poly-neddylated chain on SMURF1 can also serve as a signal for SETDB1 or other proteins to “read”. This is an interesting question for future investigation; (iii) SETDB1 recruitment triggers the formation of cCOMPASS (a SMURF1-PDK1-SETDB1 tri-complex), which facilitates AKT membrane anchorage and T308 phosphorylation for full AKT activation; (iv) SMART1, a PROTAC-based SMURF1 degrader is discovered and validated as an effective anticolon cancer agent in both *in vitro* cell culture settings and *in vivo* mouse xenograft models; (v) the “PTM two-hit” strategy was proposed and successfully tested in the case of SMART1-AR12 combination with a synergistic anticancer activity in both mouse xenograft models and human PDX models. Future studies will tell whether it is only a special case or has a broad application. Figure 1 summarizes the major findings of this study, as outlined above. Collectively, this study validates SMURF1 as an attractive target for colon cancer, particularly in cases harboring KRAS mutations.
